# Effects of sevoflurane anaesthesia on radioligand binding to monoamine oxidase-B *in vivo*

**DOI:** 10.1016/j.bja.2020.08.052

**Published:** 2020-10-06

**Authors:** Katarina Varnäs, Sjoerd J. Finnema, Peter Johnström, Ryosuke Arakawa, Christer Halldin, Lars I. Eriksson, Lars Farde

**Affiliations:** 1 Department of Clinical Neuroscience, Center for Psychiatry Research, Karolinska Institutet and Stockholm County Council, Stockholm, Sweden; 2 PET Science Centre, Precision Medicine and Biosamples, R&D Oncology, AstraZeneca, Karolinska Institutet, Stockholm, Sweden; 3 Perioperative Medicine and Intensive Care, Section for Anesthesiology and Intensive Care Medicine, Karolinska Institutet and Karolinska University Hospital, Stockholm, Sweden

**Keywords:** brain imaging, monoamine oxidase-B, positron emission tomography, sevoflurane, volatile anaesthetics

## Abstract

**Background:**

The molecular actions underlying the clinical effects of inhaled anaesthetics such as sevoflurane and isoflurane are not fully understood. Unexpected observations in positron emission tomography (PET) studies with [^11^C]AZD9272, a metabotropic glutamate receptor 5 (mGluR5) radioligand with possible affinity for monoamine oxidase-B (MAO-B), suggest that its binding is sensitive to anaesthesia with sevoflurane. The objective of the present study was to assess the effects of sevoflurane anaesthesia on the binding of [^11^C]AZD9272 and of [^11^C]*L*-deprenyl-D_2_, a radioligand selective for MAO-B in non-human primates (NHPs).

**Methods:**

Altogether, 12 PET measurements were conducted with a high-resolution research tomograph using the ligands [^11^C]AZD9272 or [^11^C]*L*-deprenyl-D_2_ in six cynomolgus monkeys anaesthetised with sevoflurane or ketamine/xylazine.

**Results:**

The specific binding of [^11^C]AZD9272 and [^11^C]*L*-deprenyl-D_2_ was markedly reduced during anaesthesia with sevoflurane compared with ketamine/xylazine. The reduction was 80–90% (*n*=3) for [^11^C]AZD9272 and 77–80% (*n*=3) for [^11^C]*L*-deprenyl-D_2_.

**Conclusions:**

Sevoflurane anaesthesia inhibited radioligand binding to MAO-B in the primate brain. The observation of lower MAO-B binding at clinically relevant concentrations of sevoflurane warrants further exploration of the potential role of MAO-B related mechanisms in regulation of systemic blood pressure during anaesthesia.

**Editor's key points**

• The molecular mechanisms underlying the clinical effects of inhaled anaesthetics are not fully understood.
• Positron emission tomography (PET) using two radioligands interacting with monoamine oxidase-B (MAO-B) were used to determine the impact of sevoflurane anaesthesia on this target in non-human primates.
• Sevoflurane anaesthesia inhibited radioligand binding to MAO-B in the primate brain at clinically relevant concentrations.
• These data suggest a potential role for MAO-B in anaesthetic effects on systemic blood pressure during anaesthesia that warrants further study.

Inhaled anaesthetics act primarily by binding to conformation-dependent amphiphilic cavities within signalling proteins of membrane-bound ion channels or ligand-gated ion channels and receptors.^1^, ^2^, ^3^, ^4^ Based on mutant animal models and structural analysis using high-resolution imaging techniques, such single or multiple binding sites of routinely used inhaled and intravenous anaesthetics have been identified within inhibitory and excitatory ligand-gated receptors and channels.^5^^,^^6^ Inhaled anaesthetics such as sevoflurane, desflurane, and isoflurane dose-dependently reduce neuronal excitability primarily by enhancing inhibitory γ-amino butyric acid (GABA)-receptor signalling while suppressing excitatory glutamate-dependent synaptic transmission.

Unlike propofol, inhaled anaesthetics have affinity (over a range of clinically relevant concentrations) for an array of protein receptors within the central and peripheral nervous systems.^7^ Such promiscuous properties serve as the basis for interactions with neuronal functions that are unrelated to anaesthetic action but with potential risk for side-effects.

As a tool for identification of subcellular targets for anaesthetic agents, molecular imaging using positron emission tomography (PET) provides a suitable approach to explore further the neurochemical properties of anaesthetics in intact tissue *in vivo* with clinically relevant exposures.^8^ As such, PET studies can provide critical insights into the effects of anaesthetics on brain metabolism and perfusion in humans.^9^, ^10^, ^11^ Moreover, PET studies of isoflurane-, sevoflurane-, or propofol-anaesthetised human subjects have shown enhanced radioligand binding to GABA type A (GABA_A_) receptors, confirming involvement of these receptor binding sites in the actions of inhaled anaesthetics.^12^^,^^13^ Interestingly, a study correlating regional cerebral anaesthetic effects with receptor density further describes the involvement of GABA_A_ receptors in the actions of propofol, but to a lesser extent for isoflurane.^10^ This observation supports the view that multiple molecular targets contribute to the anaesthetic actions of inhaled anaesthetics.

The PET radioligand [^11^C]AZD9272 was initially developed for imaging of metabotropic glutamate receptor 5 (mGluR5).^14^^,^^15^ An unexpected observation was that the specific signal for [^11^C]AZD9272 binding was conspicuously lower in non-human primates (NHPs) anaesthetised with sevoflurane compared with NHPs anaesthetised with ketamine/xylazine or in awake human subjects. These observations may suggest that [^11^C]AZD9272 binds to sites that are shared by inhaled anaesthetics. In addition to having high affinity for mGluR5, it has recently been demonstrated that AZD9272 displays significant (∼90% of specific binding) non-mGluR5-related binding that is sensitive to monoamine oxidase-B (MAO-B) inhibitors.^16^ These findings lead to the hypothesis that sevoflurane inhibits the binding of [^11^C]AZD9272 to MAO-B.

The objective of the present study was to gain further insight into the unexpected observations supporting an effect of inhaled anaesthetics on MAO-B binding in brain. Initial PET studies were conducted using [^11^C]AZD9272 in NHPs anaesthetised with sevoflurane or ketamine/xylazine. Subsequently, based on our recent observation of a shared binding site for AZD9272 and MAO-B compounds,^16^ the binding of the MAO-B radioligand [^11^C]*L*-deprenyl-D_2_^17^ was also examined in NHPs anaesthetised with sevoflurane or ketamine/xylazine. Studies were conducted in cynomolgus monkeys based on their suitability as a model for predicting drug-induced receptor occupancy in humans.

## Methods

### Radiochemistry

[^11^C]AZD9272 was prepared as described^14^ by palladium-mediated ^11^C-cyanation of the aryl bromide precursor, 5-(3-bromo-5-fluorophenyl)-3-(5-fluoropyridin-2-yl)-1,2,4-oxadiazole. [^11^C]*L*-deprenyl-D_2_ was prepared as described,^18^ by *N*-methylation of the desmethyl *L*-deprenyl-D_2_ precursor using [^11^C]methyl triflate at room temperature. Injected radioactivity was 112–212 MBq for [^11^C]AZD9272 and 153–167 MBq for [^11^C]*L*-deprenyl-D_2_.

### PET studies

The study was approved by the Animal Research Ethical Committee of the Northern Stockholm Region (Dnr N185/14), and was performed according to the ‘Guidelines for planning, conducting and documenting experimental research’ (Dnr 4820/06–600) of Karolinska Institutet and the ‘Guide for the Care and Use of Laboratory Animals’.^19^ Three male and three female cynomolgus monkeys (aged 8 [6–10] yr; weighing 7.6 [6.8–9.3] kg; mean [range]) were included in the study. The NHPs were housed in the Astrid Fagraeus Laboratory, Karolinska Institutet, Solna, Sweden. In all studies anaesthesia was induced by intramuscular injection of ketamine hydrochloride (∼10 mg kg^−1^; Ketaminol® vet; Intervet, Stockholm, Sweden ).

Altogether, 12 PET measurements were performed in six NHPs as summarised in Table 1. All measurements were performed at the PET Center at Karolinska Institutet, Stockholm, Sweden. PET measurements with [^11^C]AZD9272 were performed in three NHPs (NHP #1–3) on two experimental occasions under anaesthesia maintained either by inhalation anaesthesia with sevoflurane 3.0 vol% in oxygen and medical air or by infusion of a mixture of ketamine hydrochloride (4 mg kg^−1^ h^−1^) and xylazine hydrochloride (0.4 mg kg^−1^ h^−1^; Rompun® Vet.; Bayer, Leverkusen, Germany) i. v. After induction of anaesthesia, animals were mechanically ventilated using oxygen and air (Dräger Infinity Delta [Dräger, Lubeck, Germany] ventilator for studies with [^11^C]AZD9272; Dräger Zeus [Dräger] ventilator for studies with [^11^C]*L*-deprenyl-D_2_) at an end-tidal carbon dioxide fraction of 4–5 vol% and an inspired oxygen fraction of 0.3–0.4. Body temperature was maintained by Bair Hugger – Model 505 (Arizant Healthcare Inc., Eden Prairie, MN, USA) and monitored with an oesophageal thermometer. Heart and respiration rates were continuously monitored during the experiment. The NHP was observed continuously during the PET experimental days.

**Table 1:** Description of PET studies conducted using the radioligands [^11^C]AZD9272 or [^11^C]*L*-deprenyl-D_2._

Radioligand	NHP #	NHP body weight (kg)	Anaesthesia	Sevoflurane concentration (vol%)	Injected radioactivity (MBq)
[^11^C]AZD9272	1, 2, 3	7.8, 7.2, 7.0	Ketamine/xylazine	–	131–206
	1, 2, 3	7.6, 8.6, 6.9	Sevoflurane	2.5 and 2.9∗	112–212
[^11^C]*L*-Deprenyl-D_2_	4, 5, 6	7.0, 9.3, 6.8	Ketamine/xylazine	–	156–167
	4, 5, 6	7.0, 9.3, 6.8	Sevoflurane	3.4–3.8	153–163

∗Data not available for NHP#1. NHP, non-human primate; PET, positron emission tomography.

For studies with [^11^C]AZD9272, PET measurements performed in the same NHP were conducted at least 6 weeks apart. For each of three additional NHPs (NHP #4–6), two PET measurements with [^11^C]*L*-deprenyl-D_2_ were undertaken on the same day, including an initial measurement during sevoflurane anaesthesia and a subsequent measurement during ketamine/xylazine anaesthesia as described above (Table 1).

At each experimental session and after induction of anaesthesia, the NHP head was immobilised with a head fixation system,^20^ and PET measurements were conducted using a high-resolution research tomograph (HRRT; Siemens Molecular Imaging, Knoxville, TN, USA). Dynamic images were reconstructed as described^21^ using three-dimensional ordinary Poisson ordered subset expectation maximisation including modelling of the system's point spread function. In each PET measurement a sterile physiological phosphate buffer (pH=7.4) solution of the radiotracer was injected as a bolus into a sural vein over ∼5 s. List-mode data were acquired continuously for 123 min in [^11^C]AZD9272 PET studies and for 93 min in [^11^C]*L*-deprenyl-D_2_ PET studies based on previous investigations using these tracers.^14^^,^^15^^,^^17^

Arterial blood was sampled using an automated blood sampling system (ABSS) during the first 3 min of each PET measurement. Subsequently, arterial blood samples (1–3 ml) were manually drawn at 3, 4, 5, 8, 15, 30, 45, 60, 75, and 90 min after injection of [^11^C]AZD9272, and at 5, 15, 30, 45, 60, and 90 min after injection of [^11^C]*L*-deprenyl-D_2_. After centrifugation, 0.2–1.5 ml plasma was pipetted and plasma radioactivity was measured in a well counter. In addition, samples were taken directly from the ABSS at 0.5, 1, 1.5, 2, and 2.5 min for cross-calibration with the well counter and for determination of the plasma/blood ratio.

The fraction of radioactivity corresponding to unchanged radioligand in plasma was determined from arterial blood samples collected at 5, 15, 30, 45, 60, 75, and 90 min after injection of [^11^C]AZD9272 and at 2, 5, 15, 30, 45, 60, and 90 min after injection of [^11^C]*L*-deprenyl-D_2_ as described.^22^ The unbound fraction of radioligand in plasma was analysed using ultrafiltration.^23^

### Data analysis

**Fig 1:**
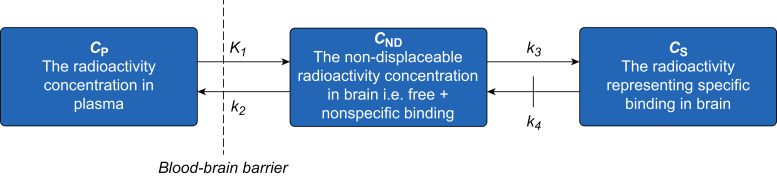
Two-tissue compartment model used to interpret regional brain radioactivity after intravenous radioligand injection. *K*_1_, *k*_2_, *k*_3_, and *k*_4_ are first order rate constants. For irreversibly bound radioligands, dissociation from the target protein is assumed to be negligible.

Regions of interest (ROIs) for evaluation of radioligand binding in the brain were manually delineated on T1-weighted magnetic resonance images acquired for the individual NHPs as described.^15^^,^^16^ All PET data were analysed using the metabolite-corrected arterial plasma curve as the input function, and were interpreted based on kinetic compartment theory assuming two tissue compartments.^24^^,^^25^ The compartments correspond to non-displaceable (free and non-specifically bound) and specifically bound radioligand, with concentrations *C*_ND_(*t*) and *C*_S_(*t*), respectively (Fig. 1). *C*_P_(*t*) corresponds to tracer concentration in plasma, and *K*_1_, *k*_2_, *k*_3_, and *k*_4_ are rate constants, where *K*_1_ and *k*_2_ describe the influx and efflux of radioligand across the blood–brain barrier and *k*_3_ and *k*_4_ describe radioligand transfer between the non-displaceable and specifically bound compartments.

Data for [^11^C]AZD9272 were analysed using the two-tissue compartment model for quantification of reversibly bound radioligands. Although the model parameter of interest for quantification of specific radioligand binding is the ratio of *k*_3_/*k*_4_, reasonable estimates of *k*_3_/*k*_4_ could not be derived with this model. Instead the more robust parameter total distribution volume (*V*_T_),^26^ expressed in terms of the rate constants according to equation (1), was used as the measure of [^11^C]AZD9272 binding.

(1) $$V_{T}=\frac{K_{1}}{k_{2}}\left(1+\frac{k_{3}}{k_{4}}\right)$$

For irreversibly bound radioligands such as [^11^C]*L*-deprenyl-D_2_, radioligand dissociation from the target protein is assumed to be negligible.^25^ Thus *k*_4_ can be constrained to zero, reducing the number of rate constants to three (*K*_1_, *k*_2_, *k*_3_; Fig. 1). The parameter *K*_1_/*k*_2_∗*k*_3_ (λ*k*_3_) yields reproducible estimates for [^11^C]*L*-deprenyl-D_2_ binding to MAO**-**B,^17^^,^^27^ and was selected as the outcome measure for analysis of [^11^C]*L*-deprenyl-D_2_ data. All PET data analyses were performed using PMOD v. 3.6 (PMOD Technologies, Zurich, Switzerland).

The effects of general anaesthesia on radioligand binding were assessed based on differences in specific binding (i.e. *k*_3_/*k*_4_ for [^11^C]AZD9272 or *k*_3_ for [^11^C]*L*-deprenyl-D_2_) under sevoflurane or ketamine/xylazine anaesthesia conditions. Relative differences in specific binding (Δ_k–s_) for [^11^C]AZD9272 or [^11^C]*L*-deprenyl-D_2_ for sevoflurane *vs* ketamine/xylazine anaesthesia were calculated based on estimates of *V*_T_ or λ*k*_3_, respectively.

In the case of [^11^C]AZD9272, Δ_k-s_ cannot be directly calculated from *V*_T_ values without an estimate of λ (*K*_1_/*k*_2_; see equation (1)), but can be obtained using a graphical method^28^ by analysing *V*_T_ values obtained for multiple brain regions. Differences in regional *V*_T_ values for ketamine/xylazine (*V*_T, ketamine_) and sevoflurane (*V*_T, sevoflurane_) anaesthesia conditions were plotted for each NHP *vs V*_T, ketamine_ and Δ_k-s_ was obtained as described^28^ from the slope of the line according to equation (2)

(2) $$V_{\text{T},\text{ ketamine}}–V_{\text{T},\text{ sevoflurane}}=\Delta_{\text{k}-\text{s}}\left(V_{\text{T},\text{ ketamine}}–\text{λ}\right)$$

Data for 12 brain regions (anterior cingulate cortex, caudate nucleus, cerebellum, hippocampus, insular cortex, occipital cortex, prefrontal cortex, putamen, temporal cortex, thalamus, ventral midbrain, and ventral striatum) were included in the analysis.

Δ_k-s_ for [^11^C]*L*-deprenyl-D_2_ was calculated for each NHP as the relative difference in λ*k*_3_ for thalamus according to equation (3)

(3) $$\Delta_{\text{k}-\text{s}}=\;\left({\left(\text{λ}k_{3}\right)}_{\text{ketamine}}–{\left(\text{λ}k_{3}\right)}_{\text{sevoflurane}}\right)/{\left(\text{λ}k_{3}\right)}_{\text{ketamine},}$$

where (λ*k*_3_)_ketamine_ and (λ*k*_3_)_sevoflurane_ are λ*k*_3_ parameter estimates for ketamine/xylazine and sevoflurane anaesthesia conditions, respectively.

## Results

Anaesthesia was maintained during PET data acquisition at an end-tidal sevoflurane concentration of 2.5–3.8 vol% (Table 1), typically adjusted to changes in heart rate and systemic blood pressure. No anaesthesia-related adverse effects on vital parameters, oxygen saturation or oesophageal temperature were observed. Free (unbound) fractions for radioligand in plasma were similar in NHPs anaesthetised with ketamine/xylazine or sevoflurane (0.22–0.32 *vs* 0.21–0.25 for [^11^C]AZD9272, and 0.17–0.23 *vs* 0.19–0.21 for [^11^C]*L*-deprenyl-D_2_). The rate of radiometabolism for [^11^C]AZD9272 was consistently more rapid for sevoflurane (63–79% parent radioligand remaining at 60 min) than for ketamine/xylazine anaesthesia (85–89% parent radioligand at 60 min), whereas the rate of [^11^C]*L*-deprenyl-D_2_ radiometabolism was similar for both anaesthesia conditions (7–14% parent radioligand at 60 min; Supplementary Fig. S1).

**Fig 2:**
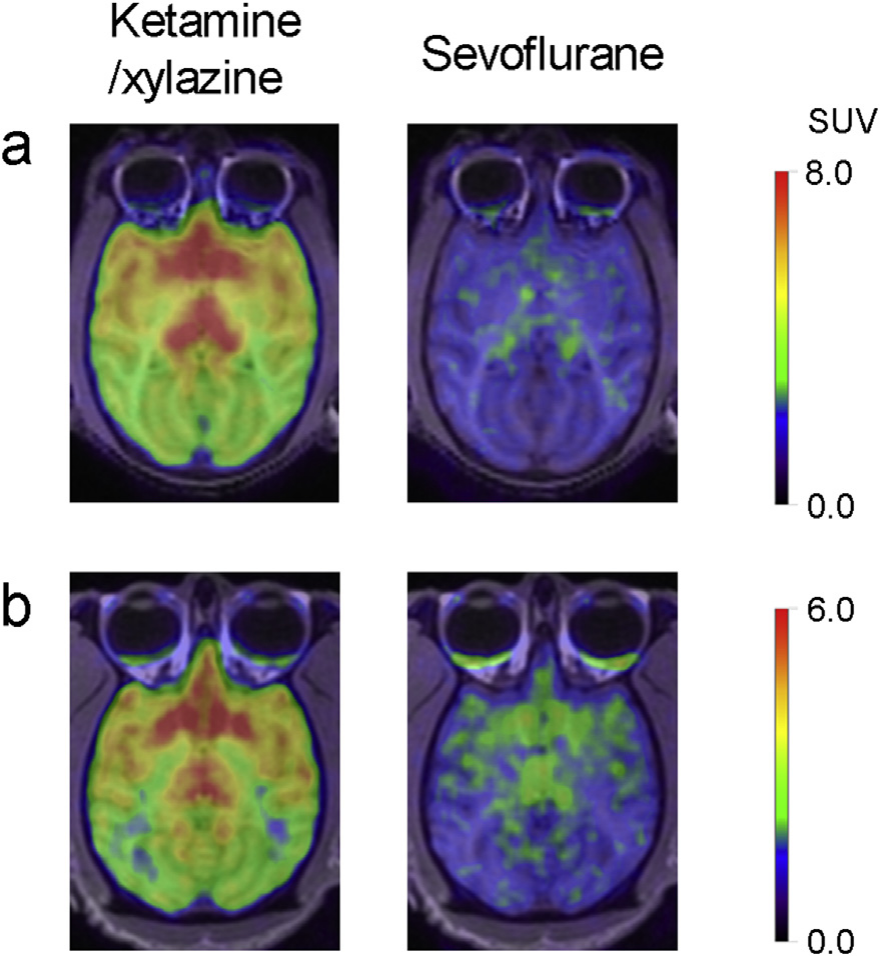
PET images for [^11^C]AZD9272 (a) and [^11^C]*L*-deprenyl-D_2_ (b) in non-human primates (NHP #3 and #4, respectively) anaesthetised with ketamine/xylazine or sevoflurane. Average images from 27 to 93 min after radioligand injection. Image intensity is presented as standardised uptake value (SUV). NHP, non-human primate; PET, positron emission tomography.

**Fig 3:**
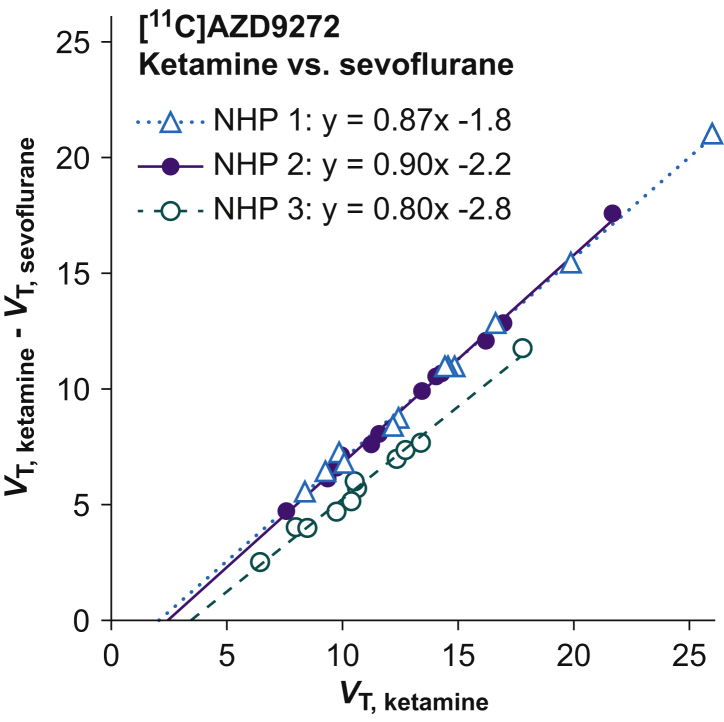
Graphical analysis of the difference in regional specific binding for the radioligand [^11^C]AZD9272 in non-human primates anaesthetised with ketamine/xylazine or sevoflurane. The relative difference in specific binding was calculated as described^28^ from the slope of the line. *V*_T, ketamine_ and *V*_T, sevoflurane_ represent the total distribution volume for ketamine/xylazine or sevoflurane anaesthesia, respectively. NHP, non-human primate.

In all brain regions, specific binding of the radioligands [^11^C]AZD9272 and [^11^C]*L*-deprenyl-D_2_ was markedly lower for NHPs anaesthetised with sevoflurane than with ketamine/xylazine (Table 2; Fig. 2a and b; Supplementary Fig. S2). The differences in *V*_T_ values for [^11^C]AZD9272 obtained with ketamine/xylazine or sevoflurane were calculated for the 12 brain regions (see Methods) and plotted *vs* the regional *V*_T_ values obtained with ketamine/xylazine anaesthesia. The relative differences (Δ_k–s_) estimated by the slope of the lines obtained in this graphical analysis were 80–90% (*n*=3; Fig. 3). The corresponding values calculated based on estimates of λ*k*_3_ for [^11^C]*L*-deprenyl-D_2_ in thalamus were 77–80% (*n*=3).

**Table 2:** Regional estimates of total volume of distribution (*V*_T_, ml cm^−3^) for [^11^C]AZD9272 and λ*k*_3_ (ml cm^−3^ min^−1^) for [^11^C]*L*-deprenyl-D_2_ in non-human primates anaesthetised with ketamine/xylazine or sevoflurane. Values are presented as mean (range; *n*=3).

Brain region	*V*_T_ [^11^C]AZD9272		λ*k*_3_ [^11^C]*L*-Deprenyl-D_2_	
	Ketamine/xylazine	Sevoflurane	Ketamine/xylazine	Sevoflurane
Caudate nucleus	17 (13–20)	4.8 (4.1–5.7)	0.40 (0.39–0.42)	0.09 (0.08–0.11)
Cerebellum	9.3 (8.0–10)	3.1 (2.6–4.0)	0.22 (0.20–0.23)	0.06 (0.05–0.06)
Prefrontal cortex	9.2 (8.5–9.7)	3.5 (2.8–4.5)	0.25 (0.24–0.26)	0.06 (0.06–0.07)
Putamen	11 (9.7–12)	4.2 (3.7–5.0)	0.28 (0.27–0.29)	0.07 (0.06–0.08)
Thalamus	15 (13–17)	4.4 (3.8–5.3)	0.34 (0.33–0.35)	0.07 (0.07–0.08)
Ventral midbrain	13 (11–14)	3.9 (3.5–4.5)	0.26 (0.24–0.27)	0.05 (0.05–0.06)

## Discussion

Anaesthesia has been identified as a potential confounder in PET studies of experimental animals.^29^ Unexpected observations with [^11^C]AZD9272, a PET radioligand originally developed for imaging cerebral mGluR5, suggest that its binding is sensitive to volatile anaesthetics. In addition, we recently reported that radiolabelled AZD9272 recognises MAO-B as its main binding site in primate brain.^16^ The effect of volatile anaesthetics may thus reflect inhibition of [^11^C]AZD9272 binding to MAO-B. Our results here corroborate the observation of low binding of [^11^C]AZD9272 within primate brain during sevoflurane anaesthesia. In addition, PET imaging using the MAO-B-selective radioligand [^11^C]*L*-deprenyl-D_2_ showed markedly lower binding for anaesthesia with sevoflurane compared with that using ketamine/xylazine. These observations support the view that sevoflurane affects similar binding sites as the radioligands known to bind to MAO-B.

Inhaled anaesthetics have been proposed to act on multiple signalling proteins,^7^ thus raising the question of specificity of sevoflurane binding to MAO-B. Although inhaled anaesthetics have been frequently used in NHP PET studies, available data have so far not shown evidence major effects of sevoflurane anaesthesia on the binding parameters for other protein targets such as [^11^C]raclopride binding to the dopamine D_2_ receptor^30^ or [^11^C]AZ10419369 binding to the serotonin 5-HT_1B_ receptor.^31^ Moreover, radioligand binding to the serotonin transporter has been reported to be similar in PET studies of awake NHPs and NHPs anaesthetised with isoflurane.^32^ These observations support the notion that the observed effect of sevoflurane on radioligand binding to MAO-B is not a generic effect on a broad array of receptors and enzymes.

Although reports of the effects of currently used anaesthetics on MAO-B activity are lacking, there is evidence supporting MAO-B inhibitory activity in tissue homogenates exposed to ether or chloroform.^33^ Moreover, inhibition of MAO has been implicated as a potential mechanism for the depression of venomotor responses induced by halothane.^34^ These observations suggest that binding to MAO-B may be a feature shared by volatile anaesthetics. Systemic blood pressure reduction is a common response during sevoflurane anaesthesia or treatment with MAO inhibitors. Although the blood pressure lowering effects of these agents are incompletely understood, both MAO inhibitors and volatile anaesthetics have been suggested to induce hypotension by depressing sympathetic activity and peripheral vascular tone.^35^, ^36^, ^37^ Our observation of reduced MAO-B binding at clinical doses of sevoflurane raises the possibility that MAO-B may be involved in the blood pressure lowering effects of volatile anaesthetics.

Sevoflurane is a commonly used anaesthetic in PET studies of experimental animals. Although sevoflurane dose is ideally constant across PET measurements, the anaesthetic concentration may need to be adjusted according to the physical condition of the animal. In previous studies of NHPs undergoing PET imaging during sevoflurane anaesthesia, sevoflurane concentration has varied significantly (up to three-fold) between experimental sessions of the same study.^30^ The present observation that radioligand binding to MAO-B is sensitive to sevoflurane anaesthesia suggests that this anaesthetic may not be optimal for PET imaging of this binding site in NHPs.

Sevoflurane anaesthesia has been found to induce global reductions in cerebral blood flow compared with the awake state,^9^ suggesting that the observation of lower radioligand binding during sevoflurane anaesthesia could partly reflect the sensitivity of binding parameters to cerebral blood flow. However, although the rate constants *K*_1_ and *k*_2_ in the equations for the main parameters of interest (*V*_T_ and λ*k*_3_) are functions of blood flow, their ratio, *K*_1_/*k*_2_, is predicted to be flow-independent. Assessment of [^11^C]AZD9272 binding was based on *V*_T_, a parameter representing tracer tissue:plasma partitioning at steady state, which does not reflect changes in blood flow.^38^ Nevertheless, binding of irreversible ligands such as *L*-deprenyl may show blood flow dependence if the rate of binding is fast relative to delivery from plasma.^27^ For this reason, to reduce the rate of trapping and sensitivity to blood flow, a metabolically stable deuterium-substituted analogue of *L*-deprenyl was used as a radioligand in the present study.^17^ Only minor variation in *K*_1_ was observed for NHPs anaesthetised with sevoflurane or ketamine/xylazine (Supplementary Tables S1 and S2). Thus, the observed differences in radioligand binding are not likely to reflect anaesthesia-related changes in cerebral blood flow.

The parameter λ (*K*_1_/*k*_2_) represents a measure for non-specific partitioning of tracer between brain tissue and plasma and is proportional to the free fraction of the tracer in plasma.^26^ The free fraction in plasma could be expected to differ if the anaesthetic agent modifies plasma protein binding of the radioligand. However, plasma protein binding measurements conducted at the time of PET examination indicated similar radioligand free fractions during the different anaesthetic conditions. In addition, λ values for [^11^C]AZD9272 obtained by graphical analysis using sevoflurane as inhibitor (2.1–3.5 ml cm^−3^) were consistent with those previously obtained for this radioligand (1.7–3.3 ml cm^−3^).^15^ These observations support that the measured differences reflect anaesthetic effects on specific radioligand binding rather than on non-specific partitioning of the tracer.

Sevoflurane anaesthesia inhibited binding of both radioligands, [^11^C]AZD9272 and [^11^C]*L*-deprenyl-D_2_. Although *L*-deprenyl is known to be an inhibitor of MAO-B,^39^ it remains to be evaluated whether this feature is shared by AZD9272. Given the markedly different chemical structures of AZD9272, sevoflurane, and *L*-deprenyl, competitive inhibition of radioligand binding seems unlikely, which suggests the possibility that sevoflurane may modulate radioligand binding through an allosteric site without affecting MAO-B enzyme activity. Future studies involving *in vitro* assays of enzyme activity are required to assess possible effects of sevoflurane and AZD9272 on MAO-B activity.

In all brain regions, *V*_T_ values for [^11^C]AZD9272 were markedly lower in NHPs anaesthetised with sevoflurane than with ketamine/xylazine. In a PET measurement conducted with [^11^C]AZD9272 in a single NHP anaesthetised with isoflurane, there was a similar extent of reduction in specific binding as with sevoflurane (81% difference relative to ketamine/xylazine; Supplementary Fig. S3). However, preliminary observations in NHPs anaesthetised with propofol indicated similar *V*_T_ values as obtained during anaesthesia with ketamine/xylazine (Supplementary Table S3). On the basis of these preliminary observations, it cannot be excluded that the inhibitory effect on radioligand binding to MAO-B is limited to the class of volatile anaesthetics.

The NHP is a model species for predicting drug-induced occupancy in humans.^40^ Given that differences in MAO-B pharmacology have been reported between primates and rodents,^41^ it remains unclear whether sevoflurane affects MAO-B binding to the same extent in rodents. Further investigations are required to assess the impact of sevoflurane anaesthesia on MAO-B binding in PET studies of other experimental species.

In conclusion, the present observations support that sevoflurane anaesthesia markedly inhibits radioligand binding to MAO-B *in vivo*. Sensitivity of MAO-B binding to sevoflurane at clinically relevant concentrations indicates that sevoflurane may not be an optimal anaesthetic agent in PET studies of this binding site in experimental animals. The observation of reduced MAO-B binding at clinically relevant concentrations of sevoflurane warrants further exploration as a potential mechanism for regulation of systemic blood pressure during general anaesthesia.

## Authors' contributions

Protocol development: KV, SJF, RA, LF

Data analysis: KV, SJF

Manuscript preparation and interpretation of data: KV, LIE, LF

Conduct of study: SJF, PJ, RA, CH

Review and revision of manuscript: KV, SJF, PJ, RA, CH, LIE, LF
