# Supplementary material for: Effects of sevoflurane anaesthesia on radioligand binding to monoamine oxidase-B in vivo
Source: Br J Anaesth. 2020 Oct 6;126(1):238–44. doi: 10.1016/j.bja.2020.08.052 (PMC8258980; doi:10.1016/j.bja.2020.08.052)
Supplement: Multimedia component 1 [file mmc1.pdf]

Supplementary Material

Effects of sevoflurane anaesthesia on radioligand binding to monoamine oxidase-B *in vivo*

K. Varnäs, S.J. Finnema, P. Johnström, R. Arakawa, C. Halldin, L.I. Eriksson, L. Farde

Table S1. Regional estimates of the influx rate constant K_1 for [^{11}C]AZD9272 in non-human primates anaesthetised with ketamine/xylazine or sevoflurane. Values are presented as mean (range; *n*=3).

	K_1 (mL*cm^{-3}*min^{-1})	
Brain region	Ketamine/xylazine	Sevoflurane
CAU	0.33 (0.28-0.41)	0.33 (0.19-0.46)
CER	0.19 (0.17-0.20)	0.38 (0.35-0.43)
PFC	0.28 (0.25-0.32)	0.33 (0.15-0.48)
PUT	0.30 (0.30-0.31)	0.33 (0.19-0.46)
THA	0.27 (0.25-0.28)	0.28 (0.21-0.32)
VMB	0.20 (0.19-0.21)	0.22 (0.16-0.25)

CAU, caudate nucleus; CER, cerebellum; PFC, prefrontal cortex;
PUT, putamen; THA, thalamus; VMB, ventral midbrain.

Table S2. Regional estimates of the influx rate constant K_1 for [^{11}C]*L*-deprenyl-D2 in non-human primates anaesthetised with ketamine/xylazine or sevoflurane. Values are presented as mean (range; *n*=3).

	K_1 (mL*cm^{-3}*min^{-1})	
Brain region	Ketamine/xylazine	Sevoflurane
CAU	0.38 (0.29-0.46)	0.26 (0.23-0.33)
CER	0.19 (0.13-0.24)	0.25 (0.19-0.28)
PFC	0.28 (0.23-0.34)	0.20 (0.18-0.22)
PUT	0.37 (0.28-0.44)	0.29 (0.24-0.35)
THA	0.28 (0.18-0.37)	0.21 (0.18-0.24)
VMB	0.19 (0.13-0.25)	0.15 (0.14-0.18)

CAU, caudate nucleus; CER, cerebellum; PFC, prefrontal cortex; PUT, putamen; THA, thalamus; VMB, ventral midbrain.

Table S3. Total volume of distribution (V_T) for [^{11}C]AZD9272 in non-human primates anaesthetised with propofol (10-20 mg kg^{-1} h^{-1} iv). Values are presented as mean (range; *n*=3).

Brain region	V_T [^{11}C]AZD9272 ($mL*cm^{-3}$)
CAU	17 (11-20)
CER	7.9 (5.8-9.4)
PFC	11 (7.6-13)
PUT	11 (7.1-14)
THA	15 (10-17)
VMB	12 (7.9-16)

CAU, caudate nucleus; CER, cerebellum;
PFC, prefrontal cortex; PUT, putamen;
THA, thalamus VMB, ventral midbrain.

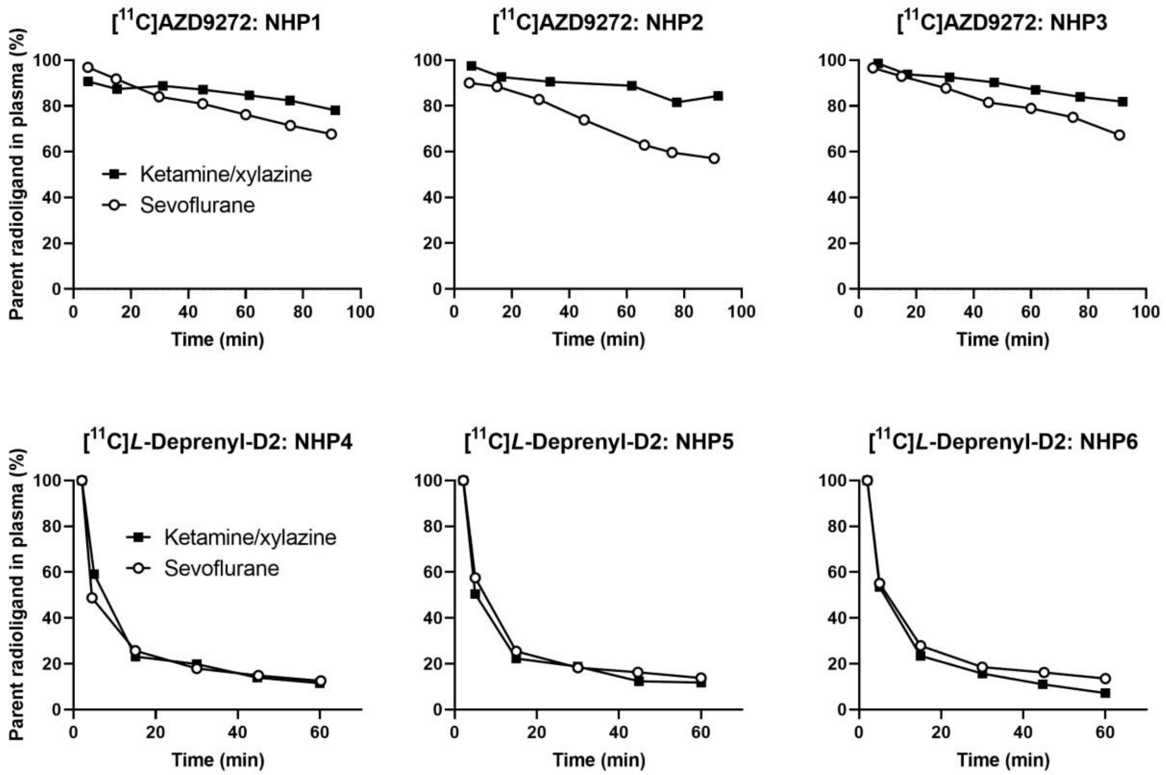

Figure S1. Percentage of unchanged (parent) radioligand in plasma determined in PET studies using [^{11}C]AZD9272 (upper panel) or [^{11}C]*L*-deprenyl-D2 (lower panel) as radioligands in non-human primates anaesthetised with ketamine/xylazine or sevoflurane.

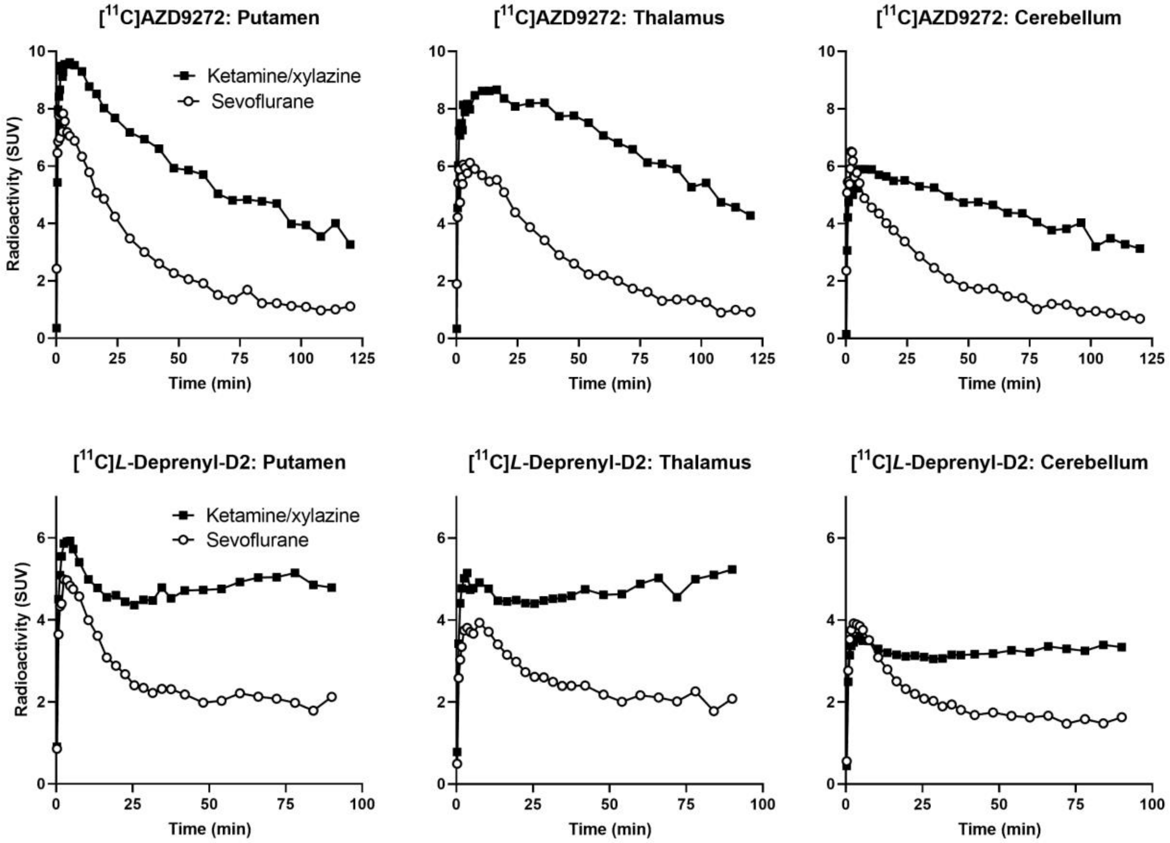

Figure S2. Time curves for regional radioactivity in PET studies using [^{11}C]AZD9272 (upper panel) or [^{11}C]*L*-deprenyl-D2 (lower panel) as radioligands in non-human primates (NHP #3 and #4, respectively) anaesthetised with ketamine/xylazine or sevoflurane.

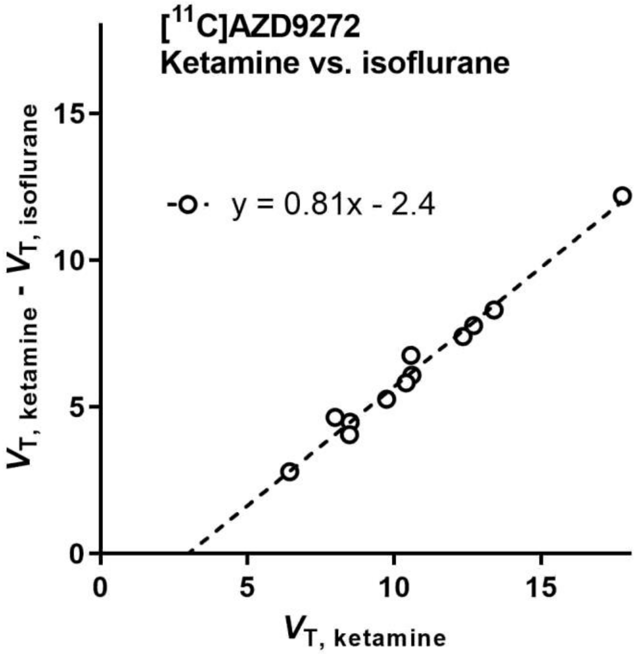

Figure S3. Graphical analysis of the difference in regional specific binding for the radioligand [^{11}C]AZD9272 in a non-human primate anaesthetised with ketamine/xylazine or isoflurane. The relative difference in specific binding was calculated as previously described (Cunningham et al., 2010) from the slope of the line. $V_{\mathrm{T,\,ketamine}}$ and $V_{\mathrm{T,\,isoflurane}}$ represent the total distribution volume for ketamine/xylazine or isoflurane anaesthesia conditions, respectively.

References

Cunningham VJ, Rabiner EA, Slifstein M, Laruelle M, Gunn RN. Measuring drug occupancy in the absence of a reference region: The Lassen plot re-visited. *J Cereb Blood Flow Metab* 2010; **30**: 46–50
